# Editorial: *Caenorhabditis elegans:* a model organism for research on medicinal plants and their metabolites

**DOI:** 10.3389/fphar.2024.1472276

**Published:** 2024-08-13

**Authors:** Dayong Wang, Xinghua Zhou, Xiaodong Zheng, Hui Dai

**Affiliations:** ^1^ Jiangsu Provincial Key Laboratory of Critical Care Medicine, Medical School, Southeast University, Nanjing, China; ^2^ School of Food and Biological Engineering, Jiangsu University, Zhenjiang, China; ^3^ Department of Food Science and Nutrition, Zhejiang University, Hangzhou, China; ^4^ Department of Chemistry, University of California Riverside Riverside, Riverside, CA, United States

**Keywords:** *Caenorhabditis elegans*, pharmacological model, pharmacological effect, medicinal plant, biological metabolite

## Introduction

This research topic aimed to explore recent developments in this area with the focus 1) to provide a comprehensive understanding of the value of model animal *Caenorhabditis elegans* for pharmacological research and 2) to highlight the usefulness of established pathological models in pharmacological study in *C. elegans*. *C*. *elegans* is a classic model animal, which has provided great contribution to the life science. Meanwhile, this animal model has been widely used for the study of pharmacology due to well-described molecular and genetic backgrounds. *C. elegans* has high sensitivity to exposure to various chemicals or drugs. With advancement for the study of biological process and mechanism of diseases, some relevant pathological models (such as Alzheimer disease (AD) and Parkinson’s disease (PD) models) have been established in *C. elegans*. These backgrounds provide the important foundation to perform systematic pharmacological study in *C. elegans* at the whole animal level.

## Pharmacological effects and mechanism of active components in medicinal plants

Largely due to the value to assess stress response and longevity, *C. elegans* has initially received the attention in the field of pharmacology. Li et al. found that phloretic acid treatment not only increased lifespan, but also improved the healthspan of nematodes. Activation of transcriptional factors of DAF-16, SKN-1, and HSF-1 and some of their downstream targets mediated this beneficial effect of phloretic acid treatment. Besides this, increased autophagy may be also involved in regulating the observed beneficial effect of phloretic acid treatment. These findings implied important anti-aging effect of phloretic acid. The further identification of direct targets of phloretic acid will be helpful to understand mechanism of this observed pharmacological effect.

## Identification of active metabolites in medicinal plants with function of anti-pathogen infection

*C. elegans* is an important animal model for the study of host-pathogen interaction. In *C. elegans*, both bacterial and fungal pathogen infection models have been established. Some anti-microbial genes in response to bacterial and fungal infection have been identified to reflect innate immunes response of *C. elegans*. Additionally, some molecular signaling pathways (such as p38 MAPM signaling pathway) have also been identified to regulate pathogen infection. Using bacterial infection model in *C. elegans*, Wang et al. found that paeoniflorin was able to reduce *P. aeruginosa* accumulation in body of nematodes. Meanwhile, bacterial infection induced lifespan reduction and inhibition in innate immunity could be suppressed by paeoniflorin treatment. For the underlying mechanism, it was found that paeoniflorin could obtain this anti-bacterial infection function by activating PMK-1, EGL-1, and BAR-1 signals. Moreover, paeoniflorin treatment suppressed *Pseudomonas aeruginosa* biofilm formation and motility and expressions of related virulence genes. These observations demonstrated the function of paeoniflorin treatment to extend lifespan of *P. aeruginosa* infected hosts by reducing bacterial accumulation and inhibiting bacterial biofilm formation. The combinational effect between paeoniflorin and drugs having function to kill bacterial pathogens is suggested to be further performed.

In addition to paeoniflorin, Shu et al. also found that cinnamon essential oil had antibacterial infection function. This was reflected by the beneficial effect of cinnamon essential oil treatment in increasing locomotion behavior, prolonging lifespan, reducing accumulation of *P. aeruginosa*, and increasing innate immune response in nematodes after the infection. Moreover, cinnamaldehyde (CID), the major component of in cinnamon essential oil, was identified to have the function of anti-bacterial pathogen infection. Activation of PMK-1 mediated p38 MAPK signaling pathway was found to mediate pharmacological effect of CID against bacterial infection. The obtained data implied usefulness of cinnamon essential oil and CID in inhibiting bacterial infection in the hosts.

## Pharmacological evaluation using pathological models

Using established pathological models, not only the pharmacological screening can be performed, but also the pharmacological mechanisms of drugs or compounds can be determined in *C. elegans*. High-glucose model established by adding 50 mM _D_-glucose in NGM plate can be used to reflect a pathological situation, hyperglycemia. Liu et al. used high-glucose model in *C. elegans* to indicate the beneficial effect of paeoniflorin against high-glucose toxicity in reducing lifespan. Moreover, paeoniflorin exhibited this pharmacological effect by inhibiting insulin signaling pathway, which was reflected by the decrease in expressions of *daf-2*, *age-1*, *akt-1*, and *akt-2* and the increase in expression of *daf-16*. That is, in insulin signaling pathway, paeoniflorin could activate transcriptional factor DAF-16, which further caused activation of its target of SOD-3/Mn-SOD. Molecular docking analysis implied possible binding potential of paeoniflorin to insulin receptor DAF-2 and kinases of AGE-1, AKT-1, and AKT-2. The results suggested the potential effect of paeoniflorin treatment in inhibiting high-glucose induced lifespan reduction. Nevertheless, the direct targeting of DAF-2, AGE-1, AKT-1, and AKT-2 by paeoniflorin needs the further experimental confirmation.

## Finding of novel function of traditional chinese medicines

Besides well described pharmacological functions, some Traditional Chinese Medicines may also have some other novel pharmacological effects. BuShen HuoXue (BSHX) is formula containing Chinese medicines for nourishing the kidney. In the clinical, BSHX decoction is used for treatment of premature ovarian failure by increasing estradiol level and decreasing follicle-stimulating hormone level. In *C. elegans*, Wu et al. first confirmed the pharmacological effect of BSHX in improving reproductive capacity in fertility-defective model established by treating animals with 175 mg/L bisphenol A. Moreover, BSHX had function against heat stress by activating HSF-1 and its several downstream targets including heat-shock proteins (HSP-16.1, HSP-16.2, HSP-16.41, and HSP-16.48). BSHX treatment also inhibited bisphenol A toxicity in inducing intestinal oxidative stress and in enhancing intestinal permeability. These observations support the anti-stress function of BSHX. The underlying mechanism for the association of this function with the effect of BSHX in improving reproductive capacity needs to be further examined.

## Usefulness for assessing the anthelmintic activity of components in medicinal plants

Considering that *C. elegans* is a kind of worm, it has been frequently used as a model to assess the possible anthelmintic activity of certain components in medicinal plants. Using *C. elegans* as animal model, Jato et al. observed that both proanthocyanidins and ellagitannins had significant anthelmintic activity. This activity was confirmed by the analysis in human intestinal nematodes. Meanwhile, this observed anthelmintic activity of proanthocyanidins and ellagitannins was affected by fecal fermentation. Two candidate compounds (proanthocyanidins and ellagitannins) with potential anthelmintic activity were further raised. The detailed pharmacological mechanisms for proanthocyanidins and ellagitannins to show anthelmintic activity are suggested to be further elucidated.

